# Single-Shot Glucosamine-Hyaluronic Acid Combination Injection for Knee Osteoarthritis: A Retrospective Observational Study

**DOI:** 10.7759/cureus.113120

**Published:** 2026-07-21

**Authors:** Amr M Ali, Engy S Attia, Abdullah O Abdullah, Mohsen A Ali, Mohamed S Dawaba

**Affiliations:** 1 Orthopaedics, National Bank Hospital for Integrated Care, Cairo, EGY; 2 Orthopaedic Surgery, Sultan Bin Abdulaziz Humanitarian City (SBAHC), Riyadh, SAU; 3 Anaesthesia and Pain Management, National Bank Hospital for Integrated Care, Cairo, EGY; 4 Anaesthesia and Pain Management, Ain Shams University Hospital, Cairo, EGY; 5 Physical Therapy, National Bank Hospital for Integrated Care, Cairo, EGY; 6 Computer and Communication Engineering, Alexandria University, Alexandria, EGY; 7 Medicine, Alexandria University, Alexandria, EGY

**Keywords:** glucosamine, hyaluronic acid, intra-articular injection, knee osteoarthritis, knee pain, lequesne index

## Abstract

Background

Knee osteoarthritis is a common cause of chronic pain, stiffness, and functional limitation. Many patients use oral glucosamine, but daily intake may be limited by adherence, pill burden, tolerability, and inconsistent long-term use. This study reviewed clinical outcomes after a single intra-articular glucosamine-hyaluronic acid combination injection used as a practical injection-based option in routine knee osteoarthritis care.

Methods

This retrospective observational study included 60 adult patients with symptomatic knee osteoarthritis treated at the National Bank Hospital for Integrated Care, Cairo, Egypt. All patients received a single landmark-guided intra-articular injection of a commercially manufactured, CE-marked Class III fixed glucosamine-hyaluronic acid combination medical device (Viscoart™ Forte GGP; ERC Biyoteknoloji Sanayi ve Diş Ticaret Anonim Şirketi, Istanbul, Türkiye). Outcomes were assessed using the Lequesne Algofunctional Index and Visual Analog Scale (VAS) pain scores recorded at baseline, approximately six weeks, and approximately six months. Previous oral glucosamine-use pattern, patient satisfaction, follow-up status, and documented adverse events were also reviewed.

Results

The cohort had a mean age of 59.5±6.1 years and a mean body mass index of 35.5±4.6 kg/m². Forty-one patients were female (68.3%), and 38 patients (63.3%) had bilateral knee osteoarthritis. Fifty-three patients (88.3%) completed the six-month follow-up. The mean Lequesne score decreased from 19.23±3.22 at baseline to 16.33±3.37 at six weeks and 14.02±3.31 at six months. Paired analysis showed significant improvement at six weeks (cohort N=60; t(59)=-41.36; p<0.001; here, 59 represents the degrees of freedom) and six months (completers N=53; t(52)=-20.47; p<0.001). The mean VAS pain score decreased from 7.45±1.10 at baseline to 5.70±1.43 at six weeks and 4.60±1.36 at six months (p<0.001 for both comparisons). Most patients had current, previous, or irregular prior oral glucosamine use (55/60, 91.7%). The mean satisfaction score at six months was 4.21±0.74 out of 5. Documented adverse events were mild and transient, mainly short-duration post-injection pain, mild redness, and one case of mild swelling; no joint infection or serious treatment-related adverse event was recorded.

Conclusions

In this routine-care retrospective cohort, a single landmark-guided intra-articular injection of this CE-marked glucosamine-hyaluronic acid combination medical device was associated with improved pain and functional scores over six months in patients with symptomatic knee osteoarthritis. These findings support further evaluation of this injection-based option in selected patients, especially where daily oral glucosamine use may be inconvenient or inconsistently maintained. Interpretation should remain cautious because of the single-arm design, missing six-month data in a small proportion of patients, and absence of an oral-glucosamine, hyaluronic acid-only, or usual-care comparator. The study describes outcomes of a single-shot glucosamine-hyaluronic acid injection in a general clinical environment.

## Introduction

Knee osteoarthritis is not only a radiographic diagnosis. For many patients, it becomes a daily problem of pain, reduced walking tolerance, slower movement, difficulty with stairs, and loss of confidence in ordinary activity. The global burden of osteoarthritis continues to rise, making practical nonoperative options increasingly important in routine orthopedic care [1].

Most treatment plans begin with patient education, exercise, weight reduction, physiotherapy, analgesics, and nonsteroidal anti-inflammatory drugs when suitable. Intra-articular injections and symptomatic slow-acting agents are also used in selected patients, although international guidelines differ in the strength of their recommendations for hyaluronic acid and glucosamine-containing therapies [2-4]. This difference between guidelines reflects the mixed and product-dependent nature of the evidence, and it makes cautious real-world reporting valuable.

Oral glucosamine is familiar to many patients with knee osteoarthritis. Some patients take it regularly, some stop after a short period, and others use it irregularly. This pattern is clinically relevant because a daily oral regimen can be affected by adherence, pill burden, tolerability, and patient preference. Recent reviews describe glucosamine and chondroitin as generally well tolerated in many human studies, but the reported clinical benefit remains inconsistent across trials and formulations [5,6].

A single intra-articular injection of a commercially manufactured, CE-marked Class III glucosamine-hyaluronic acid combination medical device (Viscoart™ Forte GGP; ERC Biyoteknoloji Sanayi ve Diş Ticaret Anonim Şirketi, Istanbul, Türkiye) represents a different practical approach. It should not be presented as proven superior to oral glucosamine, because that would require a direct comparative trial. Instead, it can be examined as an injection-based option used in routine care for patients who may not maintain daily oral glucosamine consistently or who prefer an office-based intervention. Human evidence for injectable glucosamine-containing regimens is limited but includes older intra-articular or parenteral studies and more recent reports on glucosamine-containing injection combinations [7,8].

This study reviewed routinely collected clinical data from an Egyptian hospital to describe pain, functional outcome, satisfaction, previous oral glucosamine-use pattern, follow-up completion, and documented adverse events after a single landmark-guided intra-articular injection of this CE-marked Class III glucosamine-hyaluronic acid combination medical device in patients with symptomatic knee osteoarthritis.

## Materials and methods

Study setting and design

This study was conducted at the National Bank Hospital for Integrated Care, Cairo, Egypt. It was designed as a retrospective observational review of routine clinical data. The injection was not assigned by a research protocol; it had been given as part of usual nonoperative orthopedic care, and the current study reviewed the documented clinical course after treatment. The reporting approach followed general recommendations for observational studies [9].

Clinical records reviewed

The reviewed records covered patients treated between 14 May 2025 and 29 October 2025. After the removal of identifying information, the available clinical data were transferred into a structured spreadsheet for analysis. The collected variables included age, sex, body mass index (BMI), injection date, affected side, radiographic grade, unilateral or bilateral disease pattern, previous oral glucosamine-use pattern, Lequesne score, Visual Analog Scale (VAS) pain score, satisfaction score, follow-up status, and adverse events.

Eligibility criteria

Patients were eligible for review if they were adults with symptomatic knee osteoarthritis who had persistent pain or functional limitation despite conservative care and had a documented baseline assessment before injection. Patients also needed documented early follow-up after the injection to be included in the main dataset. Patients were not included if the record showed secondary osteoarthritis, inflammatory arthritis, recent knee surgery, recent intra-articular knee injection, active local or systemic infection, known allergy or contraindication to any product component, or incomplete baseline assessment. Missing data at the late six-month interval were handled via complete-case analysis, omitting dropouts exclusively from the late paired calculations.

Baseline clinical assessment

Baseline review included demographic data, BMI, involved knee side, and whether symptoms were unilateral or bilateral. Radiographic severity was documented using the Kellgren-Lawrence grading system on standing knee radiographs [10]. When both knees were affected, the right and left grades were recorded separately. The clinical outcome analysis, however, was performed at the patient level because the recorded pain and functional scores reflected the patient's overall symptomatic status rather than a separate score for each knee.

Injection procedure and product

All patients received one landmark-guided intra-articular injection of a factory-manufactured, commercially prepared, and certified Class III European Medical Device (CE-marked) containing a fixed combination of sodium hyaluronate 90 mg and N-acetylglucosamine 90 mg per 3 mL syringe. This product is a factory-sealed commercial preparation requiring no local compounding or modifications prior to clinical administration. Under strict aseptic technique following local skin preparation with povidone-iodine, the anatomical landmark-guided injection was performed via a medial or lateral joint line approach depending on the treating physician's clinical judgment. Patient positioning, utilizing either a supine position with the knee extended or a sitting position with the knee flexed at 90°, was determined according to standard institutional clinical routine practice. To ensure consistent intra-articular deposition and control for technique variances, needle placement verification was confirmed via standard anatomical landmarks, loss of resistance, and free flow of injectate, ensuring safe intra-articular containment. Ultrasound guidance was not utilized.

Outcome assessment

The main functional outcome was the Lequesne Algofunctional Index. This index evaluates pain or discomfort, maximum walking distance, and activities of daily living in patients with osteoarthritis [11]. Pain intensity was assessed using the VAS [12]. Outcomes were reviewed at baseline, at a precise early follow-up window of 6±1 weeks post-injection, and at a late follow-up window of 6 months±2 weeks post-injection when clinical data were available. Standard oral paracetamol (up to 2 g/day) was permitted as rescue analgesia during the follow-up period but was strictly restricted for 48 hours preceding each formal clinical score evaluation. Patient satisfaction at six months, previous oral glucosamine-use pattern, follow-up completion, and documented adverse events were also reviewed.

Safety review

Safety data were taken from routine follow-up documentation. The review specifically looked for post-injection pain, redness, swelling, stiffness, local inflammatory reaction, infection, and any event requiring urgent intervention or hospital admission. Adverse events were described according to what was documented in the clinical record.

Ethical considerations

This study used anonymized retrospective clinical data collected during routine orthopedic care. No patient-identifying information, images, or personal identifiers were included in the dataset or manuscript. All data were extracted from existing medical records without any intervention assigned for research purposes. Prior to the administration of any invasive joint injection, standard written institutional clinical consent was obtained from all patients, outlining the routine clinical risks, benefits, and standard conservative alternatives. According to institutional policy for retrospective observational studies using de-identified data, formal IRB approval was not required. All treatments had been provided as part of standard clinical care prior to data analysis.

Statistical analysis

Continuous data were summarized as mean±standard deviation. Categorical data were summarized as number and percentage. Changes in Lequesne and VAS scores from baseline to follow-up were assessed using paired t-tests. Patients without six-month follow-up data were excluded only from the six-month paired analysis. A p-value of <0.05 was considered statistically significant.

## Results

The dataset is best read in three layers. First, this was a symptomatic routine-care knee osteoarthritis cohort. Second, most patients had already tried oral glucosamine in some form before reaching the injection decision. Third, the follow-up record allowed the assessment of early change in all patients and six-month change in most patients.

**Figure 1:**
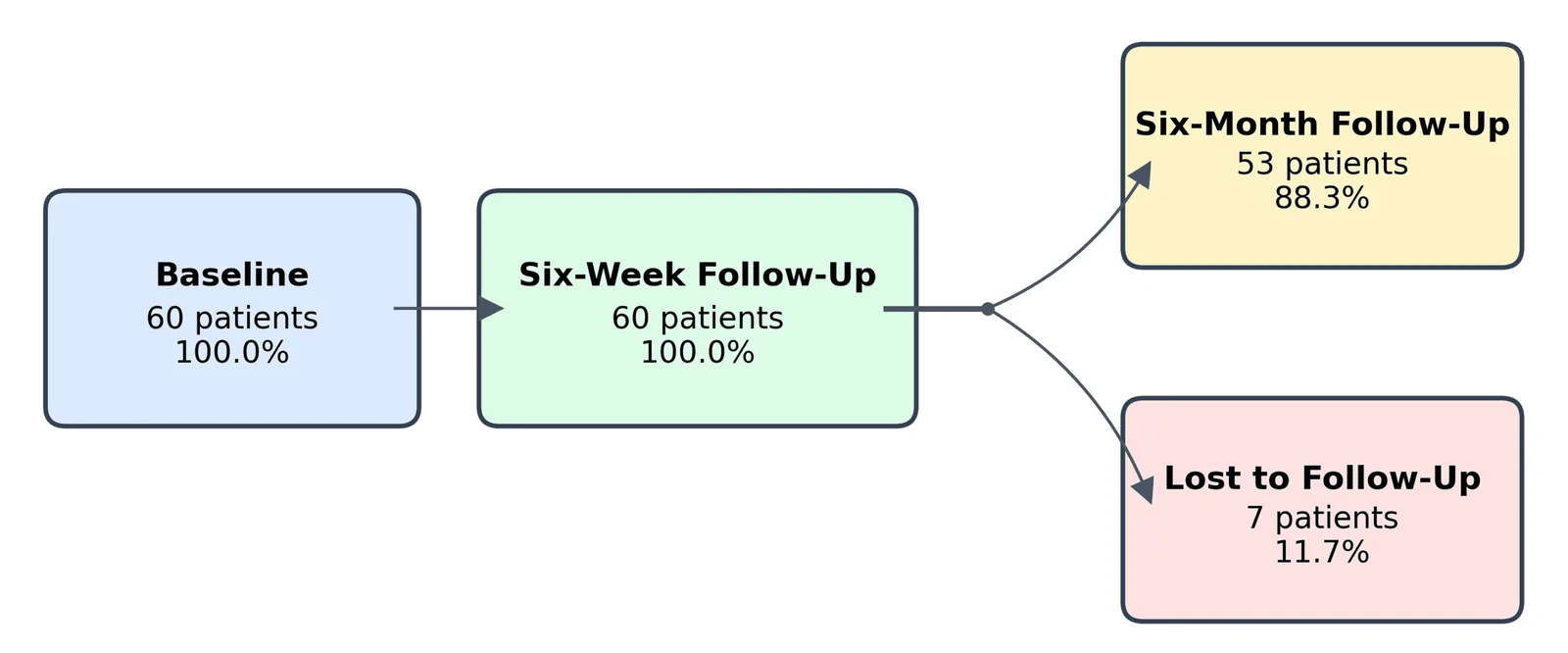
Follow-up completion flow The figure shows the number of patients available at baseline, six-week follow-up, and six-month follow-up. All 60 patients had baseline and six-week data, while 53 patients completed six-month follow-up, and seven patients were lost to follow-up.

Sixty patients were included. All 60 patients had baseline and six-week data. Six-month follow-up was available for 53 patients (88.3%), while seven patients (11.7%) did not complete the six-month assessment. The follow-up pathway is shown in Figure 1.

The mean age was 59.5±6.1 years, and the mean BMI was 35.5±4.6 kg/m². There were 41 female patients (68.3%) and 19 male patients (31.7%). Bilateral knee osteoarthritis was documented in 38 patients (63.3%). Isolated right-sided disease was documented in 12 patients (20%), and isolated left-sided disease was documented in 10 patients (16.7%). Kellgren-Lawrence grade 4 involvement was recorded in 33 patients (55%), grade 3 involvement in 30 patients (50%), and grade 2 involvement in 19 patients (31.7%). Because some patients had bilateral disease, grade involvement was counted when the grade was present in either knee. The patient profile, radiographic severity, and oral glucosamine-use pattern are summarized in Table 1.

**Table 1:** Patient profile and previous oral glucosamine use Values are presented as mean±standard deviation or number (%). KL grade involvement indicates that the grade was recorded in at least one knee. BMI: body mass index; OA: osteoarthritis; KL: Kellgren-Lawrence

Variable	Value
Total patients	60
Age, years	59.5±6.1
BMI, kg/m²	35.5±4.6
Female sex	41 (68.3%)
Male sex	19 (31.7%)
Bilateral knee OA	38 (63.3%)
Right-sided OA only	12 (20%)
Left-sided OA only	10 (16.7%)
KL grade 2 involvement	19 (31.7%)
KL grade 3 involvement	30 (50%)
KL grade 4 involvement	33 (55%)
Current oral glucosamine use	13 (21.7%)
Previous oral glucosamine use, stopped	24 (40%)
Irregular oral glucosamine use	18 (30%)
Never used oral glucosamine	5 (8.3%)

**Figure 2:**
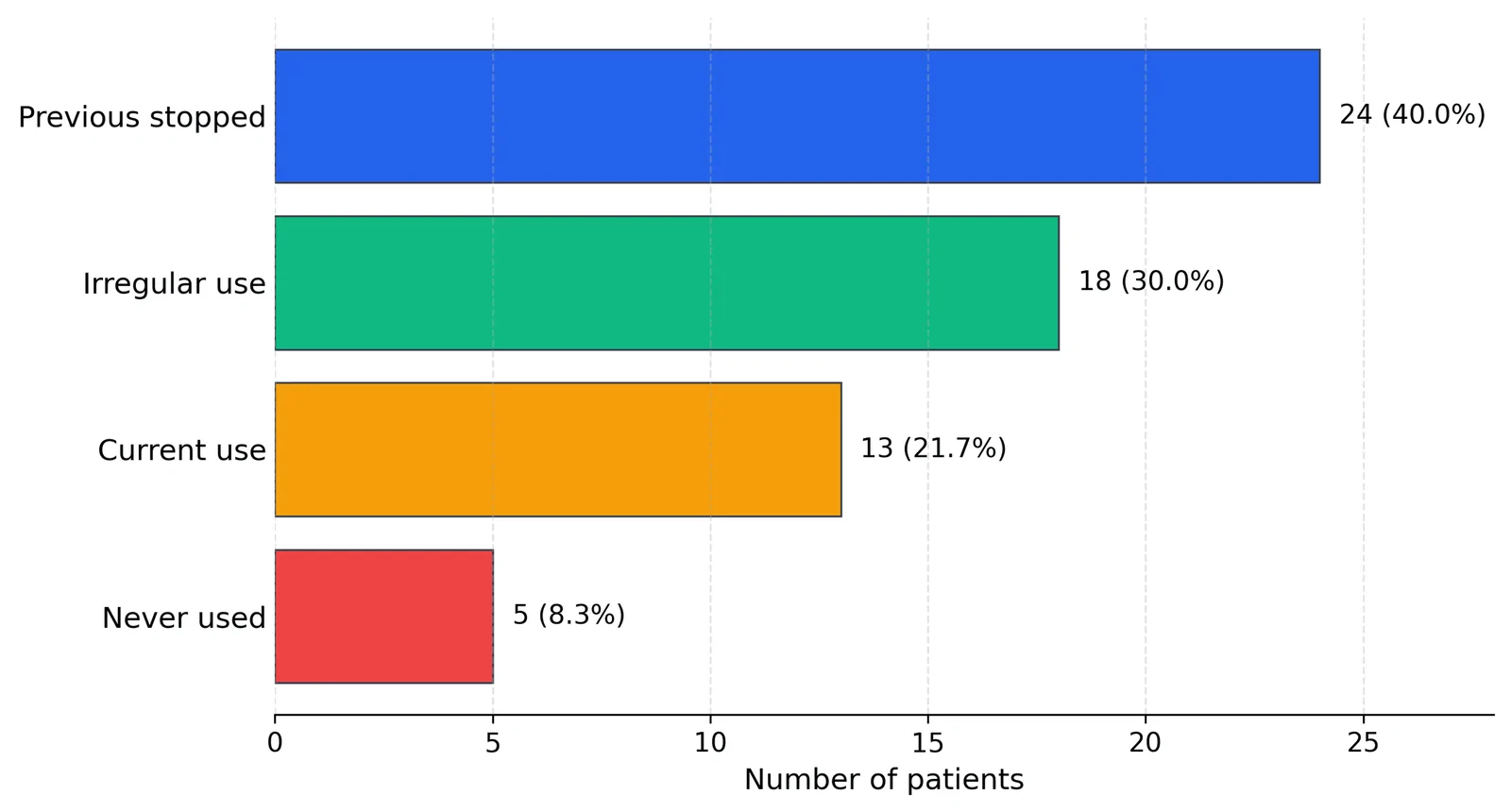
Previous oral glucosamine-use pattern The figure shows the distribution of previous oral glucosamine-use patterns in the study cohort. Most patients had either previously stopped oral glucosamine, used it irregularly, or were current users at baseline, while only five patients had never used oral glucosamine.

Previous oral glucosamine exposure was a prominent feature of the cohort. Only five patients (8.3%) had never used oral glucosamine. Twenty-four patients (40%) had used it previously and stopped, 18 patients (30%) reported irregular use, and 13 patients (21.7%) were current users at baseline. This distribution is shown in Figure 2.

Pain and function moved in the same direction after the injection. At baseline, the mean Lequesne score was 19.23±3.22. At six weeks, it decreased to 16.33±3.37, giving a mean change of -2.90 points. Paired analysis showed significant improvement at early follow-up (cohort N=60; t(59)=-41.36; p<0.001; here, 59 represents the N-1 degrees of freedom). Among the 53 patients with six-month data, the mean baseline Lequesne score was 18.85±3.12 and decreased to 14.02±3.31 at six months, giving a mean change of -4.83 points (completers N=53; t(52)=-20.47; p<0.001). 

A similar pattern was seen for pain intensity. The mean baseline VAS pain score was 7.45±1.10 and decreased to 5.70±1.43 at six weeks, giving a mean change of -1.75 points (cohort N=60; t(59)=-28.60; p<0.001). In the six-month follow-up group, the mean baseline VAS pain score was 7.34±1.09 and decreased to 4.60±1.36, giving a mean change of -2.74 points (completers N=53; t(52)=-19.49; p<0.001). 

**Figure 3:**
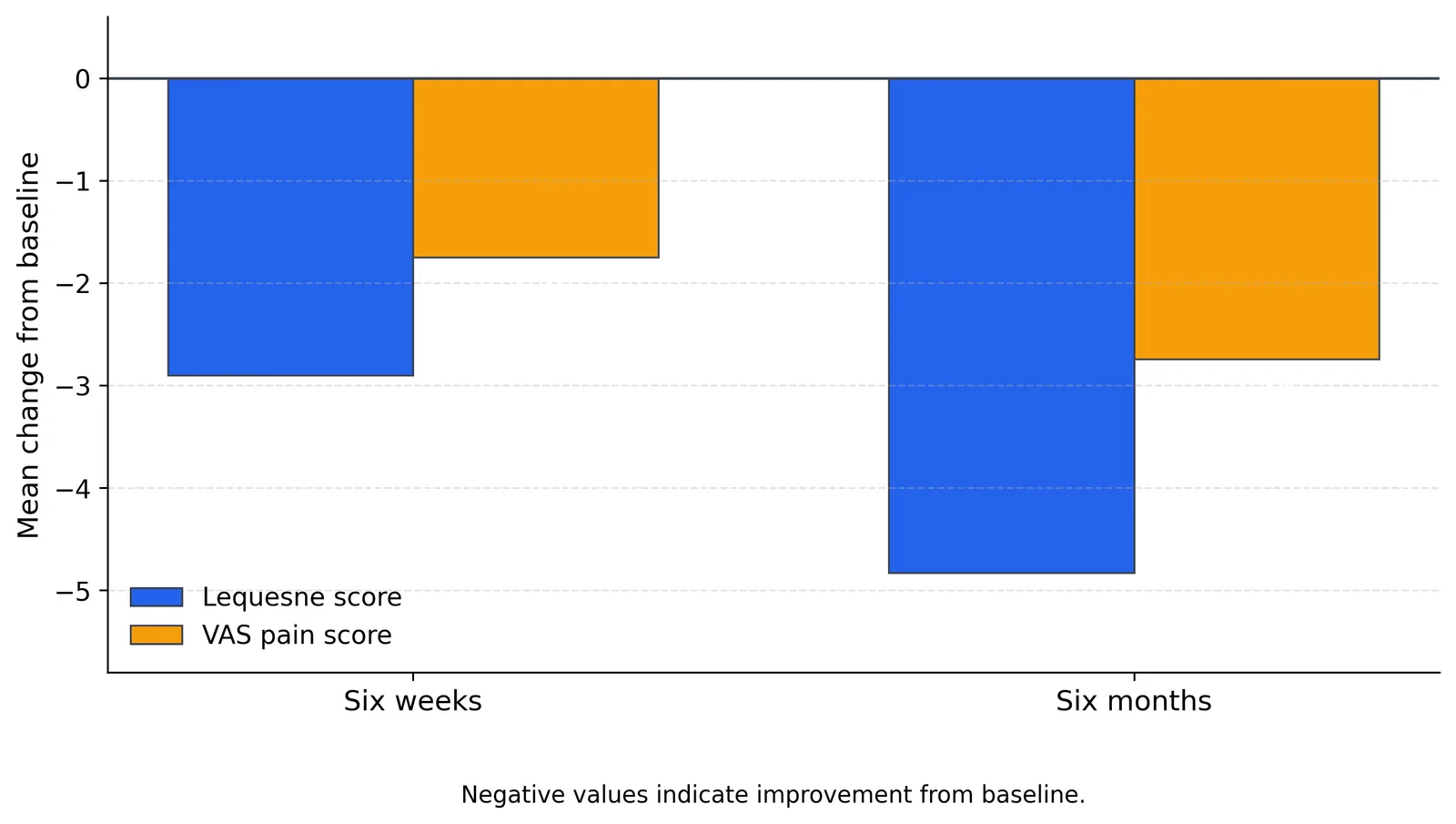
Mean change in Lequesne and VAS scores The figure shows the mean changes from baseline in Lequesne and VAS pain scores at six weeks and six months. Negative values indicate improvement from baseline. VAS: Visual Analog Scale

To evaluate the clinical relevance of these shifts, pooled effect sizes were calculated. The intervention achieved large effect sizes at six weeks (pooled Cohen's d=-0.88 for Lequesne Algofunctional Index; d=-1.37 for VAS pain scores) and at six months (pooled Cohen's d=-1.50 for Lequesne Algofunctional Index; d=-2.22 for VAS pain scores). These absolute reductions successfully crossed the standard literature benchmarks for the minimal clinically important difference (MCID), defined as 2.0 to 3.0 points for the Lequesne Algofunctional Index and 1.5 to 2.0 points for the VAS pain score [11,12]. These outcome changes are summarized in Table 2 and displayed as mean change values in Figure 3.

**Table 2:** Outcome change analysis Values are presented as mean±standard deviation unless otherwise stated. Negative mean change and negative Cohen's d values indicate clinical improvement from baseline. Six-week analysis included 60 patients, while six-month analysis included 53 patients with available follow-up data. Paired t-tests were used for baseline-to-follow-up comparisons. VAS: Visual Analog Scale

Outcome	Analysis point	Baseline	Follow-up	Mean change	Cohen's d	t-statistic	P-value
Lequesne score	Six weeks	19.23±3.22	16.33±3.37	-2.90	-0.88	t(59)=-41.36	<0.001
VAS pain score	Six weeks	7.45±1.10	5.70±1.43	-1.75	-1.37	t(59)=-28.60	<0.001
Lequesne score	Six months	18.85±3.12	14.02±3.31	-4.83	-1.50	t(52)=-20.47	<0.001
VAS pain score	Six months	7.34±1.09	4.60±1.36	-2.74	-2.22	t(52)=-19.49	<0.001

A descriptive stratification was conducted based on the clinical threshold for severe obesity to track registry trends across distinct patient phenotypes. The cohort contained 35 patients with severe obesity (BMI ≥35 kg/m²) and 25 patients with moderate or no obesity (BMI <35 kg/m²). At the six-week follow-up, the mean reduction in the Lequesne score was highly uniform between the groups (-2.89 points in the severely obese subgroup vs. -2.92 points in the non-severe subgroup). At six months, despite a higher rate of attrition in the severely obese group (six dropouts, leaving N=29 completers) compared to the non-severe group (one dropout, leaving N=24 completers), the mean functional improvement was identical at -4.83 points for both sub-cohorts. The mean six-month pain reduction followed an analogous trend (-2.76 VAS points for BMI ≥35 vs. -2.71 VAS points for BMI <35), indicating that baseline weight burdens did not alter the short-term clinical registry tracking profile.

Patient satisfaction was generally favorable among those with a six-month follow-up. The mean satisfaction score was 4.21±0.74 out of 5. A satisfaction score of 5 was reported by 20 patients (37.7%), a score of 4 by 25 patients (47.2%), a score of 3 by seven patients (13.2%), and a score of 2 by one patient (1.9%).

The safety record was limited to mild local symptoms. Short-duration post-injection pain was documented in 14 patients (23.3%), mild local redness in five patients (8.3%), and mild local swelling in one patient (1.7%). No joint infection, hospital admission, urgent surgical intervention, or serious treatment-related adverse event was documented. The satisfaction and safety profile is summarized in Table 3.

**Table 3:** Satisfaction and safety profile Values are presented as mean±standard deviation or number (%). Satisfaction was assessed among the 53 patients with available six-month follow-up data. Adverse events were extracted from routine follow-up documentation.

Variable	Result
Mean satisfaction score	4.21±0.74
Satisfaction score 5/5	20 (37.7%)
Satisfaction score 4/5	25 (47.2%)
Satisfaction score 3/5	7 (13.2%)
Satisfaction score 2/5	1 (1.9%)
Short-duration post-injection pain	14 (23.3%)
Mild local redness	5 (8.3%)
Mild local swelling	1 (1.7%)
Joint infection	0 (0%)
Hospital admission	0 (0%)
Urgent surgical intervention	0 (0%)
Serious treatment-related adverse event	0 (0%)

## Discussion

This study looked at a common outpatient situation: patients with symptomatic knee osteoarthritis who had already reached the point of considering an injection-based option. The cohort was not an early or highly selected group. Many patients had bilateral disease, high BMI, and grade 4 radiographic involvement. In that context, the observed improvement in both Lequesne and VAS scores suggests a meaningful symptomatic and functional shift, even though many patients still had residual symptoms at follow-up.

The previous oral glucosamine-use pattern gives this paper its main practical angle. Only five patients had never used oral glucosamine. Most patients were current users, irregular users, or previous users who had stopped. This does not prove that intra-articular treatment is better than oral glucosamine, and the study should not be read that way. Instead, it shows that this injection was used in a real clinical setting where daily oral glucosamine was already familiar to patients but was not always continued in a stable pattern.

The functional change is important because knee osteoarthritis patients often judge treatment by daily movement rather than by pain score alone. The Lequesne Algofunctional Index captures pain, walking distance, and activities of daily living, so improvement in this score is clinically useful when interpreted alongside the VAS pain score [11,12]. Both measures changed in the same direction at six weeks and six months, which supports the internal consistency of the recorded outcome pattern.

When clinical trends were stratified by baseline radiographic severity, patients with advanced Kellgren-Lawrence grade 4 "bone-on-bone" disease (N=33, with N=27 completing six months) demonstrated a mean six-month functional improvement of -5.11 Lequesne points and a pain reduction of -2.96 VAS points. These improvements were highly consistent with those reported by the milder grade 2/3 subgroup (N=27 with N=26 completing six months: -4.54 Lequesne points and -2.50 VAS points). Because advanced mechanical joint space narrowing is pathologically resistant to structural restoration via simple mechanical lubrication, these uniform registry tracks strongly point toward a significant contribution from placebo or contextual effects [13]. The physical act of receiving an intra-articular injection within a structured hospital setting, combined with individual patient therapeutic expectations, exerts a powerful modulating influence on subjective scoring metrics.

These findings sit within an unsettled evidence landscape. Guidelines differ in how they position hyaluronic acid and glucosamine-containing therapies, and systematic reviews continue to show that results vary according to patient selection, formulation, and study design [2-6]. This makes cautious wording essential. The present data support an observed association between treatment and improved scores, but they do not establish causality or superiority over other nonoperative options.

The injection route also deserves careful interpretation. Intra-articular therapy is more than simple delivery of a product into a joint; it occurs within a clinical encounter that includes expectation, reassurance, procedural context, and continued conservative care. Placebo and contextual effects are well recognized in knee osteoarthritis injection studies [13]. For that reason, the improvement recorded here cannot be assigned only to the mechanical effect of injection. Furthermore, regarding the clinical technique utilized, anatomical landmark-guided knee injections represent a globally established, cost-effective daily standard of care with a documented clinical accuracy rate of approximately 82% compared to 95.4% for ultrasound guidance [14]. The literature indicates that the higher anatomical precision of image guidance does not translate into superior clinical improvements in pain or physical function for routine knee osteoarthritis management [15], demonstrating that minor technique or positional variances do not alter overall patient-reported outcomes.

Evidence specifically related to injectable glucosamine-containing regimens remains limited. Older human studies reported intra-articular or parenteral glucosamine use in osteoarthritis, and more recent work has explored intra-articular glucosamine-containing combinations [7,8]. Crucially, the present study represents a completely independent retrospective analysis conducted on a distinct, separate dataset collected entirely from routine clinical records at the National Bank Hospital for Integrated Care in Cairo, Egypt. There is zero individual patient-level or analytical overlap with our previously published multicenter data from Saudi Arabia [16], reflecting a distinct institutional healthcare setting and clinical workflow. This clinical workflow utilized a factory-manufactured formulation certified as a Class III European Medical Device under international guidelines [17], ensuring standardized sterilization and composition.

To understand the distinct clinical profile of this injection, the pharmacology and pharmacokinetics of intra-articular glucosamine must be contrasted with oral administration. Oral glucosamine exhibits poor bioavailability (6.1-9.4%) due to degradation by gastrointestinal flora and extensive first-pass hepatic metabolism [18], requiring continuous daily high-dose regimens to achieve therapeutic levels in the joint. In contrast, direct intra-articular administration achieves 100% immediate local bioavailability [19]. Although the physical fluid is cleared from the joint within days, the intra-articular administration acts as a biological trigger cascade; it downregulates inflammatory mediators like interleukin-1 beta and tumor necrosis factor-alpha [20], while inhibiting COX-2 and PGE2 synthesis in chondrocytes [21]. This initiates an endogenous synthesis of high-molecular-weight hyaluronic acid by the chondrocytes, yielding a therapeutic response that persists for several months post-injection despite the rapid clearance of the initial injectate.

The safety findings were reassuring within the limits of routine documentation. Reported adverse events were mild and local, mainly short-duration post-injection pain, redness, and one case of swelling. No joint infection, hospital admission, urgent surgical intervention, or serious treatment-related adverse event was documented. This is compatible with the general safety profile described for intra-articular hyaluronic acid injections while still requiring caution because rare complications can occur and minor self-limited symptoms may be underreported in routine records [22].

As a retrospective cohort aiming to record data in a general clinical environment, the study faces a few limitations. The main limitation is the single-arm observational design. Without a hyaluronic acid-only, oral-glucosamine, or usual-care comparator, the independent contribution of each product component cannot be separated. The analysis was also patient-level, so bilateral symptoms may have influenced pain and functional scoring. Seven patients did not complete the six-month follow-up, and missing follow-up may affect the six-month estimate. Finally, the study did not include imaging follow-up, biomarkers, or long-term assessment beyond six months. While the lack of a control group limits the ability to isolate individual compound contributions, single-arm retrospective registries provide essential real-world evidence (RWE) that reflects treatment safety and performance in pragmatic, unselected, and diverse patient populations who are often excluded from rigid randomized trials [23]. This study does not suggest that global standard-of-care guidelines should be prematurely changed; rather, it documents the real-world safety and descriptive tracking of an approved protocol within a specific public hospital setting, serving as a necessary baseline that justifies the execution of future multi-arm comparative trials.

Despite these limitations, the study adds a practical routine-care observation from an Egyptian hospital cohort. A single intra-articular glucosamine-hyaluronic acid combination injection was followed by improved pain and functional scores, generally favorable satisfaction, and no documented serious treatment-related adverse event. Future studies should test this approach in controlled prospective designs, ideally comparing it with hyaluronic acid alone, oral glucosamine strategies, or usual-care pathways.

## Conclusions

In this retrospective routine-care cohort, a single landmark-guided intra-articular injection of this CE-marked Class III glucosamine-hyaluronic acid combination medical device was followed by improved pain and functional scores over six months in patients with symptomatic knee osteoarthritis. Most patients had previous, current, or irregular oral glucosamine use before injection, which supports the practical relevance of studying an injection-based option in this patient group.

These findings should be interpreted cautiously because this was a single-arm observational study without an oral-glucosamine, hyaluronic acid-only, or usual-care comparator. Future research must transition to adequately powered, multi-arm randomized controlled trials (RCTs). Specifically, prospective trials comparing this glucosamine-hyaluronic acid combination injection directly against isolated hyaluronic acid control arms, sterile saline placebos, intra-articular corticosteroids, and standard conservative management are required to definitively establish comparative efficacy, safety, long-term durability, and cost-effectiveness.
